# Challenges in Molecular Diagnostics of Channelopathies in the Next-Generation Sequencing Era: Less Is More?

**DOI:** 10.3389/fcvm.2016.00029

**Published:** 2016-09-12

**Authors:** Valeria Novelli, Patrick Gambelli, Mirella Memmi, Carlo Napolitano

**Affiliations:** ^1^Medical Genetics Unit, Fondazione Policlinico Universitario A. Gemelli, Rome, Italy; ^2^Centro Studi “Benito Stirpe” per la prevenzione della morte improvvisa nel giovane atleta, Rome, Italy; ^3^Molecular Cardiology, IRCCS Fondazione Salvatore Maugeri, Pavia, Italy

**Keywords:** genetic variants, next-generation sequencing, channelopathy genetic testing, genetic panel

## Introduction

Inherited arrhythmogenic diseases (IADs – also called cardiac channelopathies) are defined as a group of genetic diseases characterized by electrically unstable substrate in a structurally normal heart (1). Genetic testing in cardiac channelopathies has completed its transition from a research-based activity to that of a clinical genetic service. In parallel, the advancements of the sequencing technologies are providing ways to sequence several genes at a relatively low cost. This is progressively changing the approach to the genetic diagnosis of IADs. Indeed, while Sanger-based genetic testing was traditionally limited to the well characterized, most prevalent genes, next-generation sequencing (NGS) allows screening even the “minor” disease genes with very short turnaround time. This approach to genetic testing is highly efficient, but it is also generating remarkable interpretative problems mostly related to the high prevalence of variants of unknown significance (VUS), i.e., not clearly related to disease pathophysiology. This issue is relevant to several genetic diseases of the heart but is particularly evident in IADs, where the familial co-segregation analysis is hampered by the incomplete penetrance and the variable expressivity. How to design the most appropriate screening approach is challenging and it requires in-depth knowledge of the specific diseases of interest. In this Opinion article, we will review the available NGS approaches and try to outline the available strategies to optimize the performance of this genetic testing methodology.

## Genetic Testing of Inherited Arrhythmias in the ERA of NGS

Long QT syndrome (LQTS), Brugada syndrome (BrS), and catecholaminergic polymorphic ventricular tachycardia (CPVT) are the main channelopathies that can cause for sudden cardiac death (SCD) in children or young adults. In the last few years, the number of genes and genetic variants associated with these diseases has increased. For example, there are 15 known LQTS^1^ genes and at least 16 BrS genes.^2^ Importantly, however, in each disease, there are few major genes and a larger number of genes accounting for few cases each. The “minor” genes are usually poorly characterized in terms of function and pathophysiological role. As a consequence, the identification of mutations in these genes often leads to results that are difficult to interpret. Therefore, the HRS expert consensus statement on the diagnosis and management of patients with inherited arrhythmias syndromes (2) has outlined the indications for genetic testing on the basis of the epidemiological relevance of the genes and the clinical implications of genetic testing for each disease (i.e., how much the identification of the mutation can impact the clinical management).

The NGS, a massive parallel sequencing technology that revolutionized the genetic diagnostics, allows large-scale and rapid assessment of the entire human genome (3). In principle, there are three approaches that can be used: (1) whole genome sequencing (WGS), applied to sequence the entire genome, coding, and non-coding regions; (2) whole exome sequencing (WES) used to analyze only the “exome,” which represents 1% of the whole genome; (3) target resequencing panel (TRS) of genes, adopted to sequence selected gene sets/panels (4).

The first two approaches, WGS and WES, are mainly applied for research purposes, for discovery of new disease genes, while TRS is commonly used for the diagnosis in the clinical setting (5).

Recently, Pua et al. reported a comparison study applying different approaches of sequencing, such as TRS, WES, and WGS (6). Analyzing a custom panel, including 174 genes involved in inherited cardiac disease, they investigated the performances of the approaches across this set of genes. Results showed that TRS approach achieved a higher coverage (>99.8% at ≥20× read depth) compared with the other approaches (88.1 and 99.3%; WES and WGS, respectively, at ≥20× read depth). Furthermore, this approach has been reported to be faster and more affordable.

## Approach to NGS in Inherited Arrhythmias

In the pre-NGS era, the analysis of yield of genetic testing provided a clear evidence of the tight link between the severity of the clinical phenotype and rate of identified mutations. Bai et al. (7) showed a high yield of screening (64, 51, and 13% for LQTS, CPVT, and BrS, respectively) in patients with a conclusive diagnosis compared with the borderline cases (14, 13 and 2%) (7). A similar concept also applies in the NGS era. Clinicians are tempted to use the fast and efficient NGS technology as a diagnostic tool when clinical examinations are inconclusive. This can lead to the identification of a high rate of VUS, especially on minor genes. Thus, the selection of genes to be included in TRS is crucial. In general, there are three NGS strategies available:

(1) Comprehensive cardio panel: 60–180 genes covering all known genes (channelopathies and/or cardiomyopathies).
(2) Comprehensive arrhythmias panel: 20–60 genes restricted to arrhythmogenic conditions (Table 1).
(3) “Key gene” panels: few genes (3–6 genes), with high evidence, related to a specific phenotype.

**Table 1:** **Genes included in comprehensive arrhythmias panels**.

Genes	Location
ABCC9	12p12.1
ACTN2	1q43
AKAP9	7q21.2
ANK2	4q25–q26
ANKRD1	10q23.31
ANKX2.5	5q35.1
CACNA1C	12p13.33
CACNA2D1	7q21.11
CACNB2	10p12.33–p12.31
CALM1	14q32.11
CALM2	2p21
CALM3	19q13.32
CASQ2	1p13.1
CAV3	3p25.3
CTNNA3	10q21.3
DES	2q35
DSC2	18q12.1
DSG2	18q12.1
DSP	6p24.3
EMD	Xq28
GPD1L	3p22.3
HCN4	15q24.1
JUP	17q21.2
KCND3	1p13.2
KCNE1	21q22.12
KCNE2	21q22.11
KCNE3	11q13.4
KCNE5	Xq23
KCNH2	7q36.1
KCNJ2	17q24.3
KCNJ5	11q24.3
KCNJ8	12p12.1
KCNQ1	11p15.5–p15.4
LDB3	10q23.2
LMN	1q22
PDLIM3	4q35.1
PKP2	12p11.21
PLN	6q22.31
PRKAG2	7q36.1
RANGRF	17p13.1
RBM20	10q25.2
RYR2	1q43
SCN10A	3p22.2
SCN1B	19q13.11
SCN2B	11q23.3
SCN3B	11q24.1
SCN4B	11q23.3
SCN5A	3p22.2
SLMAP	3p14.3
SNTA1	20q11.21
TBX5	12q24.21
TGFB3	14q24.3
TMEM43	3p25.1
TNNI3	19q13.42
TNNT2	1q32.1
TRDN	6q22.31
TRPM4	19q13.33
TTN	2q31.2

**Figure 1:**
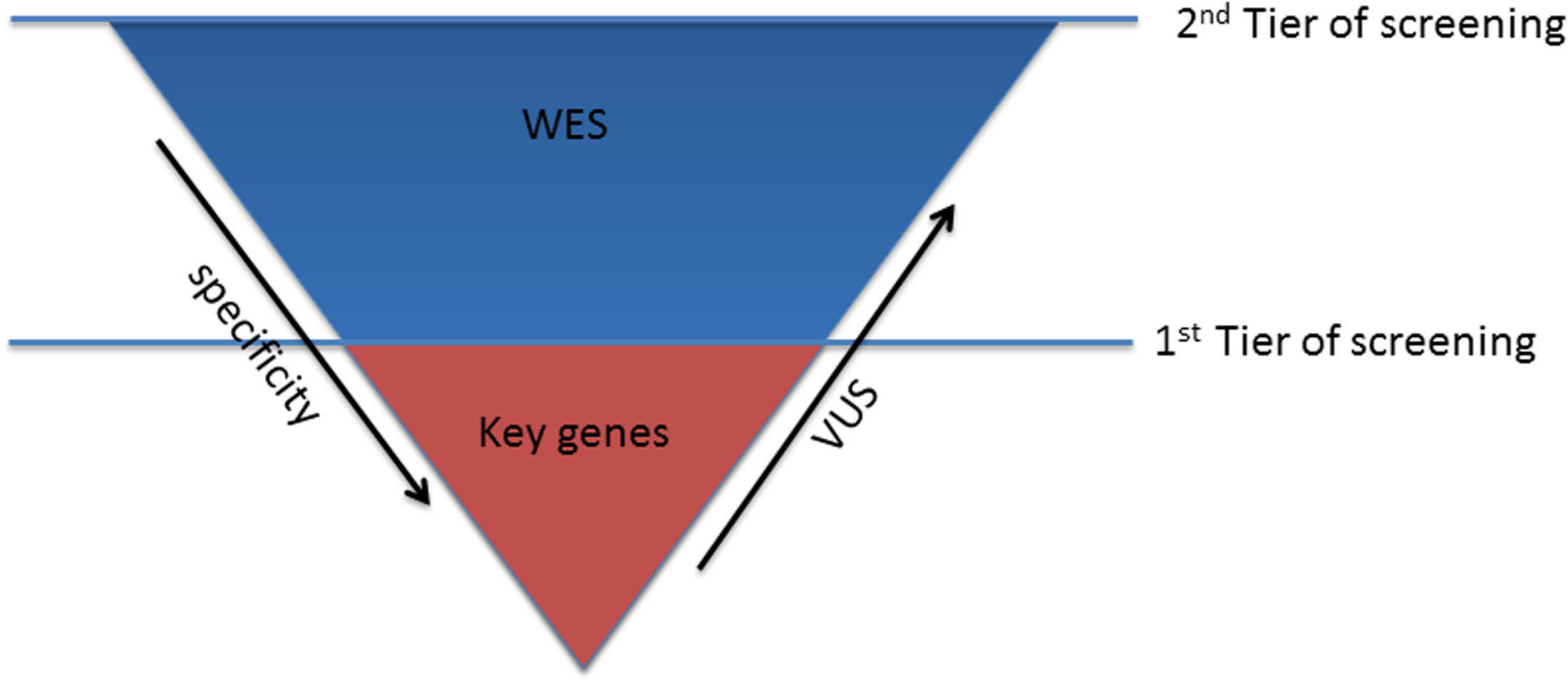
**Sequencing strategy for genetic testing**.

In patients with conclusive diagnosis, use of TRS panels with a limited set of well-characterized genes should be considered the first step to reduce the number of tests with uncertain findings (first tier) (Figure 1).

The optimal strategy in subjects, who turn out negative in the first step, is much less defined. After the exclusion of the keys genes, the second tier of screening, using WES approach, can be considered (Figure 1). This choice might be preferable over the use of comprehensive cardio panels, due to the limited evidence of the minor genes associated with channelopathies, which cannot justify the investment required for the design and production of the second larger disease-specific gene panel. Therefore, WES will guarantee the consideration of all the mutations in the minor genes that have not been unraveled yet and consider also rare genetic variants in novel genes, still unrelated with the phenotype.

Nevertheless, it is clear that there may be hurdles also in the interpretations of WES data. Independently from the screening approach, it should be considered that for diagnostic purposes the presence of (1) a clear pathophysiological link between the genetic variant and the phenotype and (2) the co-segregation within families, still remain crucial for the interpretation (8).

An example of the first tier strategy was recently reported by Millat et al. (9). Analyzing a cohort of 15 LQTS with a key panel, including only the main five genes associated with LQTS (*KCNQ1, KCNH2, SCN5A, KCNE1*, and *KCNE2*), they compared TRS and Sanger sequencing. The results showed that Sanger efficiently sequenced all the 69 exons compared with the TRS that sequenced 55/69 exons (86% of the targeted regions). NGS–TRS showed cost and turnaround time advantages over Sanger method. The study by Millat et al. highlights a very relevant problem, which is common to all NGS platforms: lack of coverage of specific regions of genes. In some cases, the problem can be particularly relevant. For example, several exons of *KCNH2*, a highly prevalent LQTS gene (~35% of patients), are not completely sequenced due to their high CG rich sequence. Thus, integration with Sanger sequencing of uncovered regions is often required with a consequence impact on costs and turnaround time.

Another interesting study on the evaluation of the first tier approach has been reported by Steffensen et al. in a cohort of 39 patients analyzed for the main genes associated with LQTS (*KCNQ1, KCNH2, SCN5A*, and *KCNE1*) (10). Results showed a high percentage of patients (17 patients; 44%) carrying variants classified as pathogenic compared with patients carrying VUS or VUS likely pathogenic (11 patients; 28%) and no alterations (13 patients; 34%).

Enlarging the screening to other seven minor genes (*ANK2, KCNJ2, CACNA1C, CAV3, SCN4B, AKAP9*, and *SNTA1*) associated with LQTS, the authors identified only three more variants, two classified as VUS and one as likely benign, demonstrating a very limited contribution, when including minor genes in the screening but a significant increase in the cost of the genetic tests.

The use of large panels, inclusive of all the minor genes may have additional limitations, as reported by Alfares et al. in patients with hypertrophic cardiomyopathy (HCM) (11). They tested over 9 years, 2,912 probands referred for clinical HCM genetic testing with different approaches: 11-gene panel, 18-gene panel, and a 50-gene pan cardiomyopathy panel. Results showed that the majority of positive tests were due to pathogenic or likely pathogenic variants in the *MYBPC3* and *MYH7* genes (83%), the two well-characterized genes routinely screened even with Sanger sequencing. Furthermore, analyzing a subset of 202 HCM patients with 18-gene panel and the pan cardiomyopathy panel, none of the probands had a causative variant outside the 18 “classic” HCM genes, suggested that use of the extended cardiomyopathy gene panel is useless for patients with HCM and should be reserved for patients with atypical clinical phenotypes (12).

## Summary and Future Developments

Next-generation sequencing technology has improved significantly over the past few years. However, there are still some limitations that need to be considered in terms of sensitivity of the uncovered regions (lower than Sanger sequencing). Moreover, the high-throughput capability is revealing itself as a double edge sword: on the one hand, it allows amazingly short turnaround time and reduced costs, but on the other hand, it reveals an increased rate of VUS and tests that are not conclusive (and therefore clinically irrelevant). A way to overcome this problem can be the implementation of a shared knowledge based on VUS. International collaborative efforts for the annotation of genetic variants are currently being explored as a mean to improve the interpretation capabilities for NGS results (13). The ClinVar database^3^ is a publicly available tool for deposition and retrieval of variant data and annotations (14). This effort is expected to support the decision on the pathogenicity of identified variants and, most importantly, to resolve the classification of VUS. Meanwhile, the most appropriate use of NGS is that of a phenotype-driven approach with sequencing panels with a limited number of well-known genes and used in patients with clear clinical indications for genetic testing. Therefore, if causative mutations are not identified on the “key” disease IAD genes, the analysis should take a “research track” with the use of WES. However, patients should be counseled accordingly.

## Author Contributions

All authors (VN, MM, PG, and CN) contributed extensively to the work presented in this opinion article.

## Conflict of Interest Statement

The authors declare that the research was conducted in the absence of any commercial or financial relationships that could be construed as a potential conflict of interest.

## Footnotes

^1^http://www.ncbi.nlm.nih.gov/books/NBK1129/

^2^http://www.ncbi.nlm.nih.gov/books/NBK1517/

^3^https://www.ncbi.nlm.nih.gov/clinvar/
